# Growth of the Cycle Life and Rate Capability of LIB Silicon Anodes Based on Macroporous Membranes

**DOI:** 10.3390/membranes12111037

**Published:** 2022-10-25

**Authors:** Galina Li, Aleksander Rumyantsev, Ekaterina Astrova, Maxim Maximov

**Affiliations:** 1 Ioffe Institute, Russian Academy of Sciences, Politekhnicheskaya st. 26, 194021 Saint Petersburg, Russia; 2 Institute of Machinery, Materials, and Transport, Peter the Great Saint-Petersburg Polytechnic University, Politekhnicheskaya st. 29, 195251 Saint Petersburg, Russia

**Keywords:** macroporous Si membranes, lithium-ion batteries, Si anodes, electrochemical impedance spectroscopy, fluoroethylene carbonate, electrochemical etching of Si

## Abstract

This work investigated the possibility of increasing the cycle life and rate capability of silicon anodes, made of macroporous membranes, by adding fluoroethylene carbonate (FEC) to the complex commercial electrolyte. It was found that FEC leads to a decrease in the degradation rate; for a sample without FEC addition, the discharge capacity at the level of *Q*_dch_ = 1000 mAh/g remained unchanged for 220 cycles and the same sample with 3% FEC added to the electrolyte remained unchanged for over 600 cycles. FEC also improves the power characteristics of the anodes by 5–18%. Studies of impedance hodographs showed that in both electrolytes (with 0% and 3% FEC, respectively) the charge transfer resistance grows with an increasing number of cycles, while Solid Electrolyte Interphase (SEI) parameters, such as its resistance and capacitance, show little change. However, the addition of FEC more than halves the overall system impedance and reduces the resistance of the liquid electrolyte and all current carrying parts as well as the SEI film and charge transfer resistances.

## 1. Introduction

**Figure 1:**
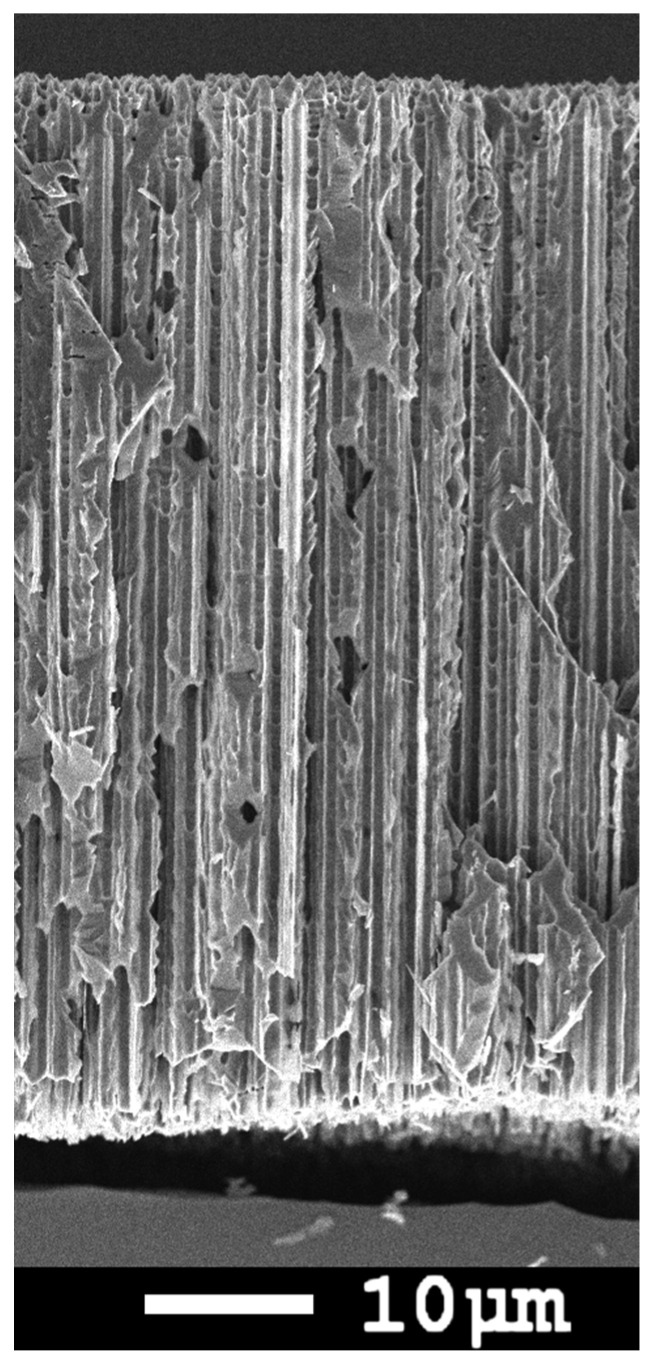
SEM image of the cross section of a macroporous membrane [10].

Silicon anodes produced by electrochemical etching of single crystalline Si wafers exhibit high specific capacities, both per mass and per sample area, and can be successfully used for lithium-ion batteries (LIB) either in the form of porous membranes [1,2] or as powders for standard slurry technology [3]. There are important parameters for the stable performance of the anodes: size of silicon elements (nanowires or nanoparticles), limitation of the inserted lithium amount, optimal current density, and charge/discharge voltage window [4,5,6]. The stability of the anodes for more than 1200 cycles in the 1000 mAh/g capacity limiting mode was demonstrated on silicon membranes with regular macropores, in which the wall thickness was ~300 nm [7]. The technology of silicon anodes was substantially simplified by photoelectrochemical etching of disordered pores in organic electrolyte, using solar grade n-Si wafers [8,9]. An example of such membranes with random macropores is shown in Figure 1. 

The main cause of silicon degradation during cycling is its cracking, which can result in complete loss of electrical contact to a part of the active material. In addition, cracking opens up new areas of silicon, on the surface of which a new layer of SEI inevitably forms. The stability of silicon anodes also strongly depends on the composition and structure of the SEI. To improve stability, conductive elements are added to silicon-containing anodes [11,12,13,14,15]) and various additives are used in the electrolyte, which mainly affect the formation of a more stable SEI [16]. It is known that FEC addition increases the cycle life by forming a protective and stable SEI layer on both the anode and cathode [17,18]. For silicon structures, a positive effect of FEC addition on both anode stability and rate characteristics has also been reported [19,20,21,22,23,24,25,26,27,28,29]. However, the effect of FEC on 3D macroporous structures consisting of high aspect ratio pores with pure Si walls has not been studied in detail. Usually, in the mentioned works, the positive effect of FEC was registered when using an electrolyte based on ethylene carbonate (EC) and diethyl carbonate (DEC) taken in the ratio (1:1). However, electrolytes of more complex composition have been used in the industry for many years. In this work, we used commercial electrolyte TC-E918 (Tinci, China), which is a 1M solution of LiPF_6_ in EC/PC/DEC/EMC mixture (ethylene carbonate, propylene carbonate, diethyl carbonate, ethyl methyl carbonate). This electrolyte is of interest, as it has been designed for cells with high voltage cathodes. The effect of FEC on commercially used complex electrolytes is not mentioned in the literature. Thus, the purpose of this work was to investigate the effect of FEC addition to the commercially used complex electrolyte and the possibility of increasing, thereby, the cyclic life, power characteristics, and Coulomb efficiency of silicon anodes made of macroporous membranes.

## 2. Materials and Methods

### 2.1. Manufacture of Macroporous Si Anodes

Electrochemical etching of solar grade n-Si (100) (by LONGI) was performed in an electrolyte consisting of a 4% HF solution in dimethylformamide and terminated by separation of the macroporous membrane from the substrate at the transition to the electropolishing mode. The technology is described in more detail in [9,10]. It should be noted that after etching of macropores, the walls between them remain single crystalline. To make the anodes, copper with a chromium sublayer was deposited on the upper surface of the membrane by vacuum thermal deposition, and then a copper layer 8–10 µm thick was electroplated. 

The electrodes for the cells with and without FEC additive were cut from the same silicon membrane and had the same area and mass, which allowed the exclusion of the influence of these parameters in the comparison. Several pairs of such samples cut from different membranes with similar parameters were studied in this work. The behavior of such pairs was well-reproduced; therefore, the characteristic data obtained for one of the pairs are presented hereafter. Two electrodes with an area of S = 0.3 cm^2^ each and an active mass (Si) of 0.93 mg were cut from an *l* = 51 μm thick membrane with a gravimetric porosity of 74% and an average wall thickness of *t* = 150 nm after application of copper contact. These electrodes were assembled into CR2032 coin-like cells, where the second electrode was a 250-µm thick lithium metal (Tob New Energy).

### 2.2. Electrochemical Measurements

The half-cells were cyclically tested in the galvanostatic mode on the CT3008W-5V10 mA bench (Neware) and 3 wt. % FEC (Aldrich) was added to TC-E918 electrolyte. The lithiation (charge) was limited to a specific capacitance *Q*_ch_ ≈ 1000 mAh/g and to a potential *U* = 10 mV relative to lithium. Such limitation of the charged capacitance allows the reduction of the amount of Li in the Li_x_Si alloy and thus a decrease in the mechanical stresses and a significantly lower degradation rate [7]. Thus, the voltage variation was in the range of 10–2000 mV. During lithiation, either capacitance or voltage limitation was triggered. For the first 20 cycles, the anodes were cycled at a current density of 0.11 A/g, then the current value was increased to 0.16 A/g (21–25 cycles), 0.22 A/g (26–30 cycles), and 0.32 A/g (31–35 cycles), which equaled the rates C/8.9, C/6.2, C/4.4, and C/3.1, respectively. From cycle 36, the current density was returned to 0.11 A/g and remained so until the end of the experiment.

### 2.3. Impedance Spectroscopy Method

Impedance measurements were performed using a modular potentiostat/galvanostat Biologic VSP in the frequency range *ν* from 100 kHz to 0.01 Hz; the amplitude of the alternating voltage was 10 mV. Our previous experiments have shown that the impedance measured in two- and three-electrode schemes has virtually no difference. The conclusion that the transition from the three-electrode cell to the two-electrode does not make visible changes in the impedance spectra was reported by the authors of [30,31]. Therefore, all measurements of impedance were carried out for the anodes on the two-electrode circuit in the CR-2032 cells.

## 3. Results and Discussions

### 3.1. Electrochemical Characteristics of Anodes

**Figure 2:**
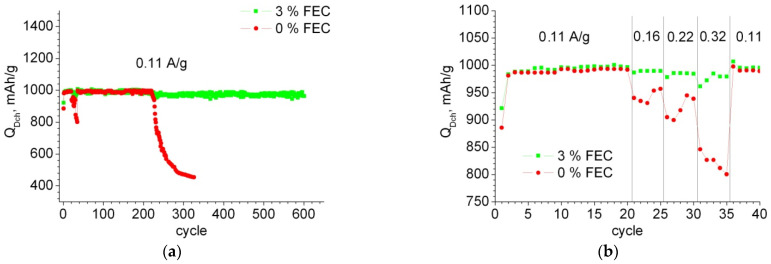
Dependence of specific discharge capacity on the cycle number for samples with 0% and 3% FEC: (**a**) throughout the experiment, (**b**) during the first 40 cycles at different currents shown in the graph.

Figure 2a shows a typical dependence of the discharge capacity on the cycle number for samples in cells with 0% FEC and 3% FEC. It can be seen that for the samples without FEC addition, the delithiation capacity starts to decrease sharply after 220 cycles, whereas for the samples with FEC, it remains at 1000 mAh/g for more than 600 cycles. Figure 2b shows an enlarged portion of Figure 2a, the first 40 cycles, where cycling was performed with a change in current. It can be seen that when the cycling current density is increased, the samples with FEC addition exhibit 5–18% higher capacitance than the samples without the addition. The increase in power characteristics with FEC addition was also noted by the authors of [28,29].

**Figure 3:**
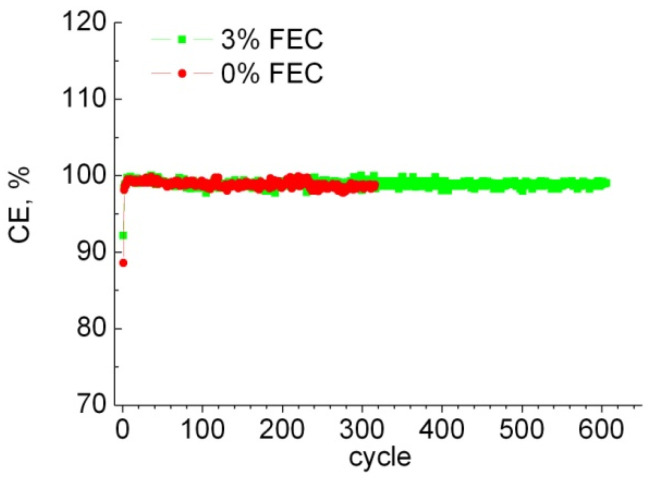
Dependence of Coulomb efficiency on the cycle number for samples with 0% and 3% FEC.

Coulomb efficiency for all samples is at 99–100%, except for the first two charge/discharge cycles (see Figure 3). Note that for the sample without additive, even after 220 cycles, the Coulomb efficiency remained at 99–100%, indicating that all of the embedded Li is extracted, while its charge capacity begins to gradually decrease. Presumably, this is due to degradation of the anode and loss of contact to some parts of the active material.

**Figure 4:**
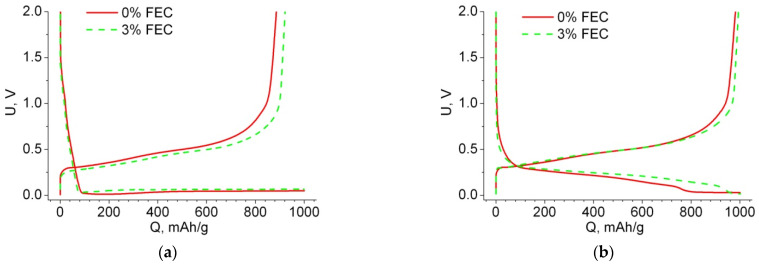
Voltage profiles of the first (**a**) and second (**b**) cycles for samples with 0% and 3% FEC.

Let us consider the features of the charge–discharge curves for macroporous anodes with single crystalline walls. Figure 4 shows the charge–discharge curves of the first two cycles for samples with and without FEC addition. The first cycle of lithiation, regardless of the FEC addition to the electrolyte, has an almost horizontal plateau characteristic of Li inserting in crystalline silicon. The delithiation curve of the first cycle shows irreversible losses; this amount of electricity was spent on the electrolyte reduction reaction and SEI formation. The amount of SEI formed can be judged by the irreversible capacitance. Figure 4a shows that for the sample with FEC, the SEI loss at the first cycle was 8%, and without the additive it was 11%. Since the design of the both cells (and anodes) is identical, except for the FEC addition, the difference in the discharge capacitances can be explained by the interaction of FEC with the electrode, the decomposition of FEC, and the incorporation of reaction products into the SEI [27]. Thus, the larger value of the Coulomb efficiency in the case of 3% FEC addition can be related to a slightly different composition of the SEI and inform a hypothesis that its thickness is smaller. SEI formation usually ends at cycle 2 (see Figure 2 and Figure 4).

**Figure 5:**
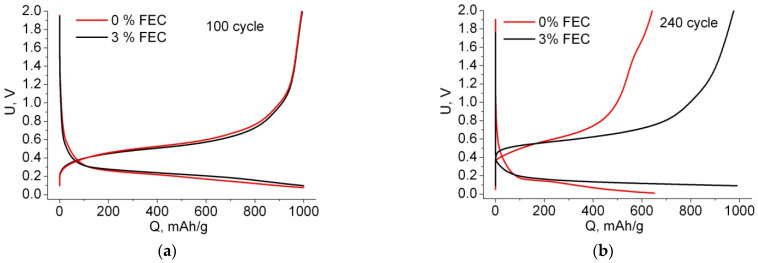
Voltage profiles with 0% and 3% FEC for cycles number 100th (**a**) and 240th (**b**).

It is known that after lithium extraction, silicon becomes amorphous, and insertion of lithium into amorphous silicon is characterized by a downward sloping line [32,33,34]. Indeed, in Figure 4b, we see that the second lithium intercalation is characterized by the sloping curve characteristic of embedding into amorphous silicon. At the end of this curve there remains a horizontal section, which indicates the insertion of Li into crystalline silicon. Note that the greater the losses in the first cycle, the greater the horizontal plateau of lithium “additional insertion” in the second cycle (compare Figure 4a,b). Therefore, for the sample without FEC addition, the length of this section is ~200 mAh/g, and for the sample with FEC, it is ~50 mAh/g. Since we are working under the conditions of charge capacity limitation, lithiation over the entire Si wall thickness does not occur during the first cycle; this process is discussed in more detail in our work [35]. With the number of cycles, the length of this plateau decreases, and by cycle 50, the charge curve becomes fully sloped (e.g., cycle 100 in Figure 5a). Note that the disappearance of this plateau for the sample with and without FEC occurs simultaneously. This suggests that the addition of FEC does not affect the rate of advance of the amorphous/crystalline silicon front (except for the second cycle, where the difference in the insertion area length into the crystalline silicon is due to the difference in irreversible capacitance at the first cycle). From this we can conclude that under limited capacitance conditions, the amorphous/crystalline silicon front penetrates to the full depth of the thin silicon walls during the first 50 cycles. The uniform descending part of the charging curve (Figure 5a) went down in voltage with each subsequent cycle, so that the voltage at which the electrode was charged to *Q*_ch_ = 1000 mAh/g has been changed, and from some cycle a potential limit 10 mV began to operate (Figure 5b). For samples without FEC, this started at cycle 220, and for samples with FEC, the voltage limitation was not observed during all cycle numbers.

**Figure 6:**
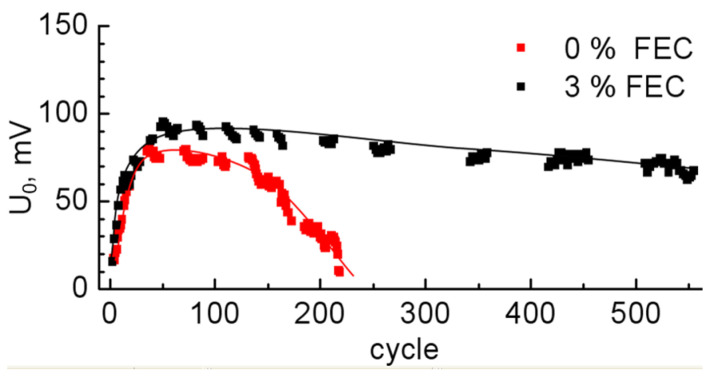
Dependence of the voltage *U*_0_ corresponding to the end point of the lithiation curve at *Q*_ch_ = 1000 mAh/g.

Figure 6 shows the graph of the voltage *U*_0_ at the end point of lithiation *Q* = 1000 mAh/g as a function of the cycle number. It can be seen that at the beginning of cycling, the voltage *U*_0_ increases, reaches a maximum, and then begins to decrease smoothly. The maximum voltage at *U*_0_ = 80–90 mV for samples with 0% and 3% FEC is observed for the 50th cycle, which on the voltage profiles corresponds to the absence of a horizontal part at the end of charge and manifests complete amorphization of the silicon wall. Since the Li insertion during these 50 cycles corresponds to the capacity of 1000 mAh/g, as the amorphous/crystalline silicon front advances deeper into the silicon wall, the x-value in the Li_x_Si compound at the surface near the border with the electrolyte becomes smaller with each subsequent cycle. As a consequence, this is reflected in the increase of the voltage *U*_0_ at the end point of the charging curve during the first 50 cycles (Figure 6). For samples without FEC addition after the 50th cycle, the rate of voltage drop with the number of cycles is much higher than for samples with 3% FEC. At cycle 220, the voltage *U*_0_ for the samples without FEC reached 10 mV, and there was a sharp decrease in the charging capacity and, consequently, discharging capacity, in the subsequent cycles. Apparently, this is due to the fact that with each cycle, due to cracking, there is a loss of contact with some part of the sample and its ohmic resistance increases. Thus, the addition of 3% FEC reduces the degradation rate and thus increases the cycle numbers with a given capacity of 1000 mAh/g by more than three times. Note also that the curve of Li insertion for samples with FEC is slightly higher in voltage than the curve for samples without additive, and, on the contrary, lower in the case of extraction (Figure 4 and Figure 5). We observed this dependence throughout all cycles (which can also be seen from the final voltage in Figure 6). This phenomenon of decreasing electrode polarization when adding FEC was also observed in other studies, for example, in [27,36].

### 3.2. Study of Anodes by Impedance Spectroscopy

**Figure 7:**
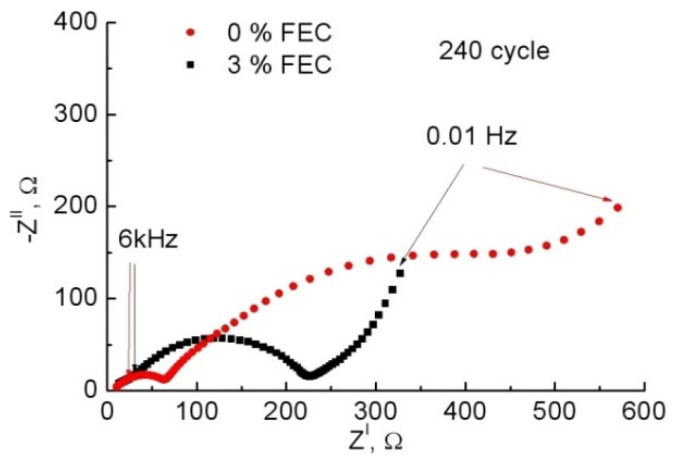
Impedance hodographs for the 240th charge cycle for samples with 0% and 3% FEC.

Figure 7 shows the impedance hodograph for anodes with 0% and 3% FEC for the 240th cycle. The charge voltage was 10 and 90 mV, for the sample without and with FEC, respectively. After a long pause of 10 h, the voltage stabilized at 0.3 V and 0.5 V, respectively. Figure 7 shows that in the case with FEC, the impedance decreased almost twofold: at 0.01 Hz it was |Z| = 350 Ohm compared to |Z| = 600 Ohm in the sample without FEC addition. It is known that the cell impedance decreases when FEC is added, regardless of the material used [21,22,24,29,37]. In the case of Si, this decrease can be up to four times [27]. For a correct comparison of impedance spectra, numerical simulations were performed using the ZView software (Scribner Associates Inc.). In order to reasonably select the electrical scheme describing the impedance hodograph changes, it was necessary to study the behavior of the system when varying either voltage or temperature. We conducted impedance measurements as a function of voltage. Lithium insertion was carried out at a current density of 0.11 A/g up to voltages of 0.5; 0.4; 0.3; 0.2; and 0.1 V and then in the voltage constant mode for 4 h. The impedance was also measured at extraction under the same conditions. In both cases the impedance at the same voltage coincided, indicating the correctness of the experiment performed. 

**Figure 8:**
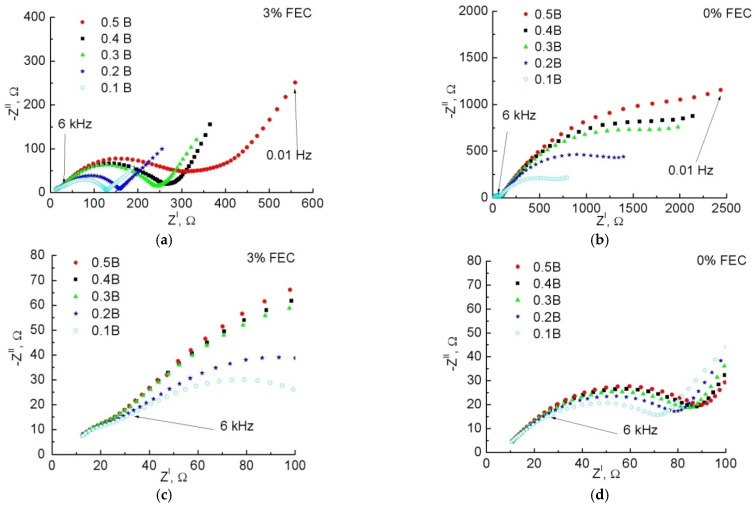
General view (**a**,**b**) and high-frequency region (**c**,**d**) of impedance hodographs at different charge voltages specified in the legend for samples with 3% FEC (**a**,**c**) and 0% FEC (**b**,**d**).

Figure 8 shows the impedance hodographs for samples with 0% and 3% FEC, recorded at different voltages. It is known that for the negative electrode, increasing the amount of inserted Li leads to a decrease in impedance [38,39,40,41,42,43,44]. Whatever the equivalent circuit, the length cut off from the abscissa axis corresponds to a purely ohmic resistance *R*_0_, which is determined by the resistance of the electrolyte and all current carrying parts. This resistance is little dependent by voltage [38]. Depending on the voltage, the high-frequency arc responsible for the SEI layer does not change, either in its shape or size [38,39,41,42]. An exception is the work [45], where partial dissolution of SEI with increasing potential in the area of active Li extraction is noted and, as a result, the formation/dissolution processes compete, which is reflected in the layer resistance. The charge transfer resistance across the electrode/electrolyte frontier decreases with decreasing voltage, and the double layer capacitance changes little [41,42,46]. This region is usually in the mid-frequency region of the spectrum and is located at lower frequencies than the arc associated with the SEI, although there are exceptions [30,40]. 

**Figure 9:**
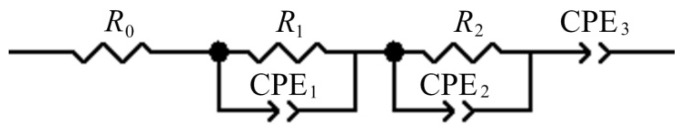
Equivalent circuit used for the impedance hodograph fitting.

From Figure 8c,d we can see that the half-circle in the high-frequency region indeed changes its shape and size little when the voltage changes, and therefore belongs to the SEI region, while the arc in the middle region of the spectrum strongly changes its diameter when the voltage changes and belongs to the double layer region. Based on the obtained spectra, the following equivalent electrical circuit was proposed, presented in Figure 9, where the CPE are constant phase elements, for which the impedance is defined as:

Z_CPE_ = *A*^−1^(*jω*)^−*n*^, (1)

In expression (1), *A* is the coefficient of proportionality and *n* is the exponent denoting the phase deviation. Depending on the value of *n*, the coefficient of proportionality acquires a certain physical meaning and dimensionality. When *n* = 1 − *ε*, it is capacitance in F, when *n* = 0 ± *ε*, resistance in Ohm^−1^, when *n* = 0.5 ± *ε*, Warburg element in Ohm × s^−0.5^, where 0 ≤ *ε* < (0.1–0.2) [47]. The first pair with parallel chain *R*_1_ and CPE_1_ corresponds to the SEI parameters, the second pair *R*_2_ and CPE_2_, to the charge transfer across the Si/electrolyte boundary and the double layer capacitance, respectively. The third component, CPE_3_, represents the Warburg impedance and characterizes the Li diffusion into Si. The initial values *A*_1_ = 10^−6^, *A*_2_ = 10^−5^, and *A*_3_ = 10^−2^ in units of s^n^/Ohm were set in the fitting program; these values correspond to those found earlier for similar structures both from our experience and from literature data [30,40]. The values of *R*_0_, *R*_1_, and *R*_2_ corresponded to the approximate intersection with the Re Z axis on the obtained impedance diagram. The values of n were set on the assumption of correspondence to the SEI and double layer regions, where *n*_1_, *n*_2_ = 1.0, and the region of Li diffusion in Si, where *n*_3_ = 0.5. None of the given initial parameters were fixed. The error of each parameter after fitting was in the range of 0.7–7%. The fitting data are given in Table 1.

**Figure 10:**
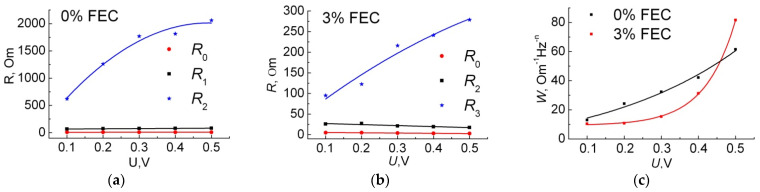
Dependences of fitting parameters *R*_0_, *R*_1_ and *R*_2_ on voltage for sample with 0% (**a**) and 3% (**b**) FEC, as well as dependence on voltage of Warburg coefficient W (**c**).

Table 1 shows that the resistance of the electrolyte, the SEI layer, and the charge transfer for the sample with FEC is less than that for the sample without additive. The voltage dependences of these parameters are shown in Figure 10a,b. Note that the values of n obtained by fitting agree very well with our notions of which region they belong to. Thus *n*_3_ = 0.4–0.55, typical for the Warburg layer, *n*_1_ = 0.62–0.67 for the SEI layer, and *n*_2_ = 0.65–0.72 for the double layer [30,47]. In our case, the deviation of the n3 value from the case of classical ideal diffusion into an infinite electrode did not exceed ±0.1, which is due to the fact that ideal structures do not take into account porosity and structure roughness, which can lead to deviation of the phase shift from π/4, i.e., from *n* = 0.5. Deviations of *n*_1_ and *n*_2_ from the ideal capacitance with *n* = 1.0 did not exceed, respectively, −0.38 and −0.35, which in real structures may be related to the composition of the SEI, such as its layered structure or porosity. 

According to numerous works when FEC is added, regardless of the electrode material used [17,26,27], it is generally accepted that FEC as a result of decomposition enriches the SEI with LiF, which is a good conductor for Li^+^ ions, thereby increasing the ionic conductivity. The SEI on silicon consists of an inner inorganic layer (i.e., Li_2_CO_3_, LiF, etc.) near the electrodes and an outer organic layer on the electrolyte side (lithium ethylene ecarbonate, polycarbonate, etc.). SEI growth, its composition, and porosity are influenced by various factors, including current density, lithiation and delithiation potential window, presence of binding components, and coatings. Additional LiF, formed by the addition of FEC, is also actively involved in the formation of SEI. Its larger amount in the SEI was recorded by the authors of [26]; as a result, a denser SEI is formed in the case of FEC. More dense SEI can prevent the penetration of small molecules (i.e., LiPF_6_, P-O and Li-O particles) to the anode surface, as well as prevent the hydrolysis of LiPF6, leading to the formation of Li_x_PO_y_F_z_ and further to Li_3_PO_4_. In addition, a large amount of LiF can protect against cracking of Si particles. According to [26], this is due to the fact that LiF, which covered the surface of Si particles, partially suppressed the volume change during cycling due to its higher elasticity.

Based on our data, *R*_0_ for the sample with FEC is 1.5–3.0 times smaller, which is probably due to greater mobility of Li^+^ ions in the electrolyte due to FEC addition, which agrees with the data obtained by the authors after 100 cycles [27]. The lower *R*_1_ is due to the increased LiF content in the SEI layer, making it denser and thinner under the condition of FEC addition [26,27]. *A*_1_ grows with increasing amount of intercalation lithium for both the sample with FEC and without addition. Presumably, it is caused by an increase in Si particles volume under Li insertion, thus the surface area increases and the SEI layer on their surface becomes thinner (*C* = *εε*_0_*S*/*d*). At the same time, as the surface area increases and the SEI thickness decreases, *R*_1_ should decrease (*R*_1_ = *ρ*_SEI_·*d*/*S*, where *ρ*_SEI_ is the SEI resistivity, Ohm·cm). For the sample without FEC, this is observed. For the sample with FEC, on the contrary, *R*_1_ slightly increases as the voltage decreases. Since the SEI layer is considered as a solid electrolyte, the values of the charge transfer resistance at the Si/SEI interface and the double layer capacitance directly depend on the physical properties of this SEI layer, its composition, and thickness. In such a case, the lower charge transfer resistance *R*_2_ in the case of FEC addition may be due to a thinner SEI layer. A significant drop in *R*_2_ with decreasing voltage is associated with a decrease in the total resistance of the electrode during Li insertion. Probably, it is the lower values of *R*_1_ and *R*_2_, as well as the presence of the electrode polarization reduction phenomenon that leads to an increase in the power characteristics of the silicon anode under conditions of 3% FEC addition (Figure 2b). The capacitances *A*_1_ and *A*_2_ in the case of 3% FEC are lower than those without FEC, which is probably due to the less porous structure of the SEI under conditions of FEC addition [26].

The third CPE_3_ chain with *n*_3_ = 0.4–0.5 simulates the low-frequency linear section. Figure 8 shows that in the low-frequency region in both samples, Z_CPE_ decreases with decreasing voltage *U*, which, according to Formula (1), corresponds to an increase in parameter *A*_3_ (Table 1). According to the law for semi-infinite diffusion, the frequency dependence of Im Z can be expressed as follows:

−Im Z = *W*/√2πν (2)

where the parameter *W* is the Warburg coefficient. The dependence of *W* on the voltage calculated by Formula (2) is shown in Figure 10c. In turn, *W* is related to the lithium diffusion coefficient in Si by the formula [40]:

*D* = (d*U*/d*Q*)^2^/2*ρ*^2^(*WS*)^2^ (3)

where *ρ*_Si_ = 2.33 g/cm^3^, the density of crystalline silicon, *S*–Si anode area, cm^2^, d*U*/d*Q*-the slope of the inclined section of the galvanostatic charging curve in the voltage range 0.3–0.01 V, Ohm × g/s.

Figure 10c shows that for both samples, *W* decreases with increasing amount of inserted Li. This agrees with the general notion of an increase in the diffusion coefficient with the intercalation of Li into the electrode, which is due to the loosening of the material as it increases in volume [41]. Let us calculate the diffusion coefficient for the studied anodes at cycle 240. Taking into account the fact that after cycle 220, for the sample without FEC, we associate the decrease of charge/discharge capacity with cracking of the sample and loss of some active material, calculation of the slope d*U*/d*Q* from Figure 5b can lead to erroneous values since the charge capacity is already distributed over a smaller active mass. Therefore, the calculation for the sample without FEC addition was not performed. According to (2), for the sample with 3% FEC, the estimated diffusion coefficient was 8 × 10^−13^ cm^2^/s, which is in order of magnitude consistent with the data in other works for Si electrodes [40,48].

**Figure 11:**
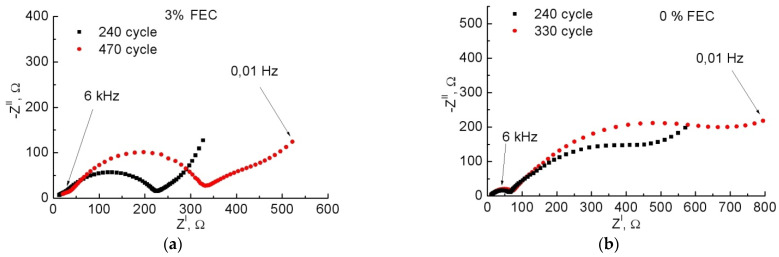
Impedance hodographs for samples with 3% (**a**) and 0% (**b**) FEC as a function of the cycle numbers.

The study of impedance hodographs at different cycles (Figure 11) showed that with increasing number of cycles for both samples, *R*_0_ increases, which seems to be associated with electrolyte aging [30,38]. *R*_1_ and *R*_2_ also increase with the number of cycles for both samples. However, *R*_1_ increases little while *R*_2_ increases much more. From this we can conclude that once SEI is formed, it changes little with the number of cycles. This is evidenced by the Coulomb efficiency, which in all our experiments is close to 99–100%, even when the anode degrades, and we see a drop in the charging and discharging capacities (lithiation and delithiation, respectively). However, material degradation, cracking, and loss of contact can lead to an increase in *R*_2_, as we see for both samples with the number of cycles. In the case of FEC, this increase is smaller, and the absolute *R*_2_ value for the sample with FEC, even for the 470th cycle, is still significantly lower than for the sample without additive for the 240th cycle, where we observe degradation. Thus, these data once again confirm the fact that with FEC addition, the anodes behave more stably and their degradation is minimal.

## 4. Conclusions

In this work, it was found that the addition of 3% FEC to the commercial electrolyte with a complex composition of carbonates improves the cyclic stability and power characteristics of silicon anodes based on macroporous membranes. For the obtained structures, it was shown that the samples with FEC retain their discharge capacity at 1000 mAh/g at a current of 0.11 A/g for more than 600 cycles, which is three times higher than for the samples without additive. When the lithiation/delithiation current was increased, the anodes with FEC added to the electrolyte showed a higher charge/discharge capacity than the samples without such an addition. The irreversible losses of the first cycle for samples with FEC decrease by 11% to 8%. The rate of anode degradation as cycling was estimated by impedance measurements after 240 cycles. The studies showed that the electrolyte resistance *R*_0_ was 1.5–3.0 times lower with the addition of FEC than without the addition. Parameters *R*_1_ and *A*_1_ are also smaller with the addition of FEC than without the addition. Changes of SEI parameters (*R*_1_ and *A*_1_) with the number of cycles are minimal both for the sample with FEC and without it. The charge transfer resistance *R*_2_ at the SEI/Si interface increases with the number of cycles, which is probably due to sample cracking and loss of contact to a part of the active material. Moreover, *R*_2_ in the case of the sample without FEC addition is at least two times higher than the values established for the samples with FEC addition. We think that cracking in the case of FEC addition occurs slower, which is likely connected with the fact that SEI has a different composition and greater ionic conductivity and is apparently more elastic. The discharge capacity per unit of the nominal electrode area is 3.1 mAh/cm^2^, which with the anode thickness of 51 μm and stability around 600 cycles is very promising for their use in LIB.

## Figures and Tables

**Table 1:** Impedance fitting data as a function of voltage.

	0% FEC					3% FEC				
*U*, V	0.1	0.2	0.3	0.4	0.5	0.1	0.2	0.3	0.4	0.5
***R*_0_**, Ohm	7.5	7.7	7.9	8.0	8.1	5.0	5.0	3.8	3.3	2.8
***R*_1_**, Ohm	66	73	76	78	82	26	27	21	19	18
***A*_1_**, μF	45	38	32	29	27	15	15	13	13	12
***n*_1_**	0.62	0.64	0.65	0.66	0.67	0.65	0.65	0.65	0.65	0.65
***R*_2_**, Ohm	620	1300	1800	1800	2100	95	120	220	240	280
***A*_2_**, μF	1400	1200	1100	1100	1100	49	43	39	45	56
***n*_2_**	0.65	0.68	0.70	0.72	0.71	0.69	0.70	0.66	0.64	0.62
***A*_3_**, Ohm^−1^·Hz^−*n*3^	0.019	0.012	0.007	0.006	0.005	0.043	0.028	0.031	0.026	0.008
***n*_3_**	0.50	0.50	0.50	0.50	0.55	0.46	0.50	0.55	0.56	0.40
***W***, Ohm/√s	10	11	15	31	82	13	24	32	42	62

## Data Availability

Not applicable.
